# Supplementary material for: Impact of b‐value on estimates of apparent fibre density
Source: Hum Brain Mapp. 2020 Mar 26;41(10):2583–95. doi: 10.1002/hbm.24964 (PMC7294071; doi:10.1002/hbm.24964)
Supplement: Supplementary file 1 — Figure S1 Example of preprocessing and analysis outputs for representative participants aged 8, 13, and 18 years old: A) raw dMRI data; B) preprocessed dMRI data; C) fibre orientation distributions (FODs) pre‐ and post‐registration to the population‐based template. Figure S2: Representative images of tract bundles extracted for statistical analysis, generated using TractSeg. The left view of the tract is presented in each case. Tracts are coloured by the direction of streamlines (red: left–right; green: anterior–posterior; blue: inferior–superior). Figure S3: AFD for simulated fibre geometries across five sampling schemes with noise. Variations to simulated intra‐axonal signal fraction and perpendicular diffusivity of the extra‐axonal space (De,⊥) were tested to compare AFD across multiple fibre geometries. Simulations were performed with noise (SNR = 50 35 and 25) with 100 Rician noise generalisations (error bars denote mean ± 2SD). Sampling schemes reflect the chosen b‐values, in s/mm2. With lower SNR (greater noise), the estimated AFD was more variable indicated by a larger spread of values, particularly at smaller intra‐axonal signal fractions. Figure S4: Dendrogram heatmap highlighting clusters of tracts which differentially describe age‐related differences in apparent fibre density (AFD) across various single‐shell b‐value sampling schemes. Heatmap colour intensity reflects range of R 2 values derived from a linear model including age and sex. A depiction of several fibre pathways in one cluster is presented on the right. Figure S5: Dendrogram heatmap highlighting clusters of tracts which differentially describe age‐related differences in apparent fibre density (AFD) across various multi‐shell b‐value sampling schemes. Heatmap colour intensity reflects range of R 2 values derived from a linear model including age and sex. Note: datasets with two diffusion weightings (including the b = 0) were processed using the multi‐shell multi‐tissue FBA framework, thus res [file HBM-41-2583-s001.docx]

1. **Supplementary material**
   1. **Supplementary information**

All of the children included in the study were typically developing. None of the children reported a previous clinical diagnosis of ADHD or learning disabilities (based on parent report). Out of the children included, we only collected handedness information for 59/78 participants (76%). Of these children, 11 were left-handed (18%) and 48 were right-handed (62%).

- 1. **Supplementary figures**


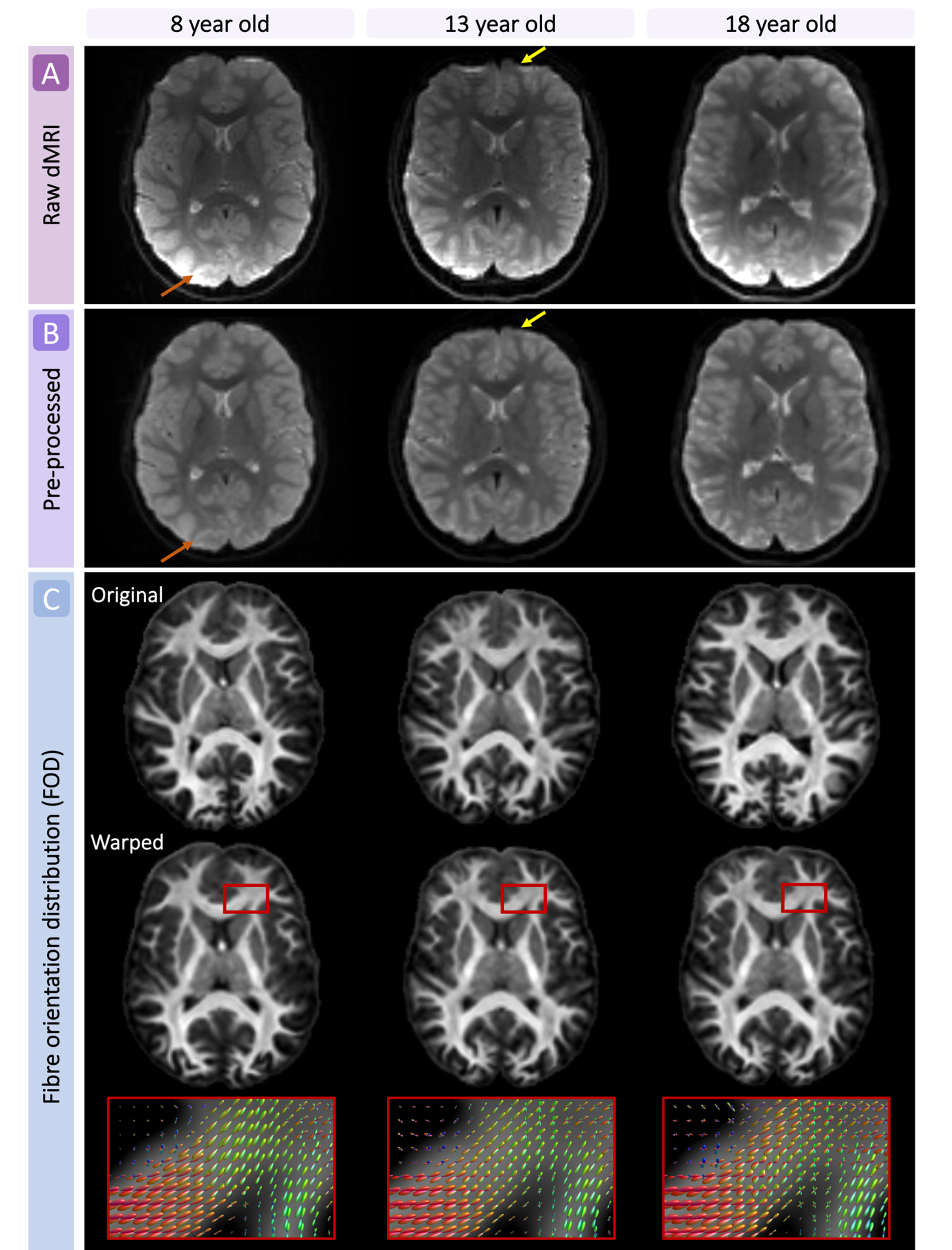


**Figure S1:** Example of pre-processing and analysis outputs for a representative participant aged 8, 13 and 18 years old. A) Raw dMRI data; B) pre-processed dMRI data; C) fibre orientation distributions (FODs) pre- and post-registration to the population-based template.

**
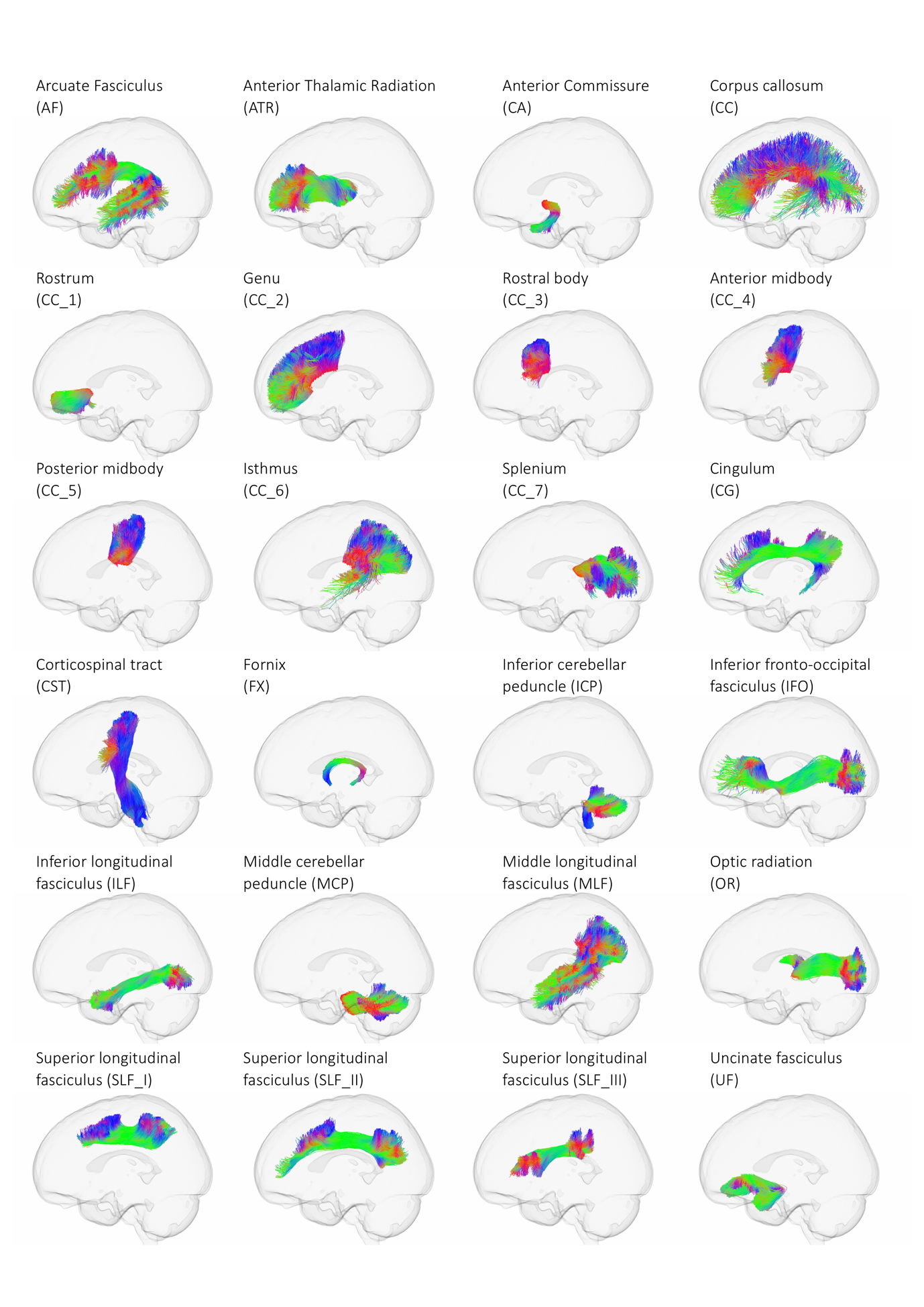
**

**Figure S2:** Representative images of tract bundles extracted for statistical analysis, generated using TractSeg. The left view of the tract is presented in each case. Tracts are coloured by the direction of streamlines (red: left-right; green: anterior-posterior; blue: inferior-superior).


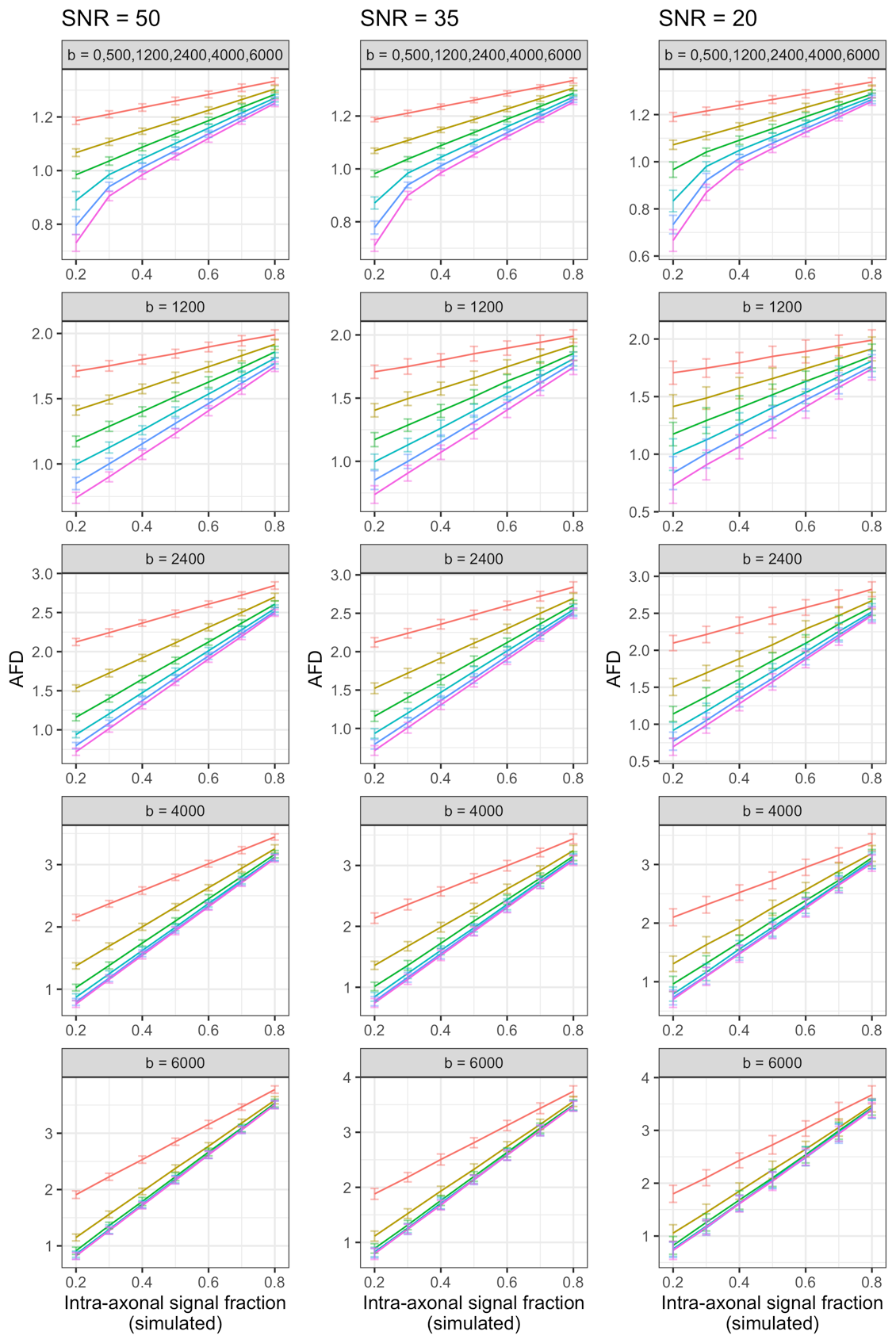


**Figure S3:** AFD for simulated fibre geometries across five sampling schemes with noise. Variations to simulated intra-axonal signal fraction and perpendicular diffusivity of the extra-axonal space ($D_{e,\perp}$) were tested to compare AFD across multiple fibre geometries. Simulations were performed with noise (SNR=50; 35; and 25) with 100 Rician noise generalisations (error bars denote mean ± 2SD). Sampling schemes reflect the chosen b-values, in s/mm^2^. With lower SNR (greater noise), the estimated AFD was more variable indicated by a larger spread of values, particularly at smaller intra-axonal signal fractions.


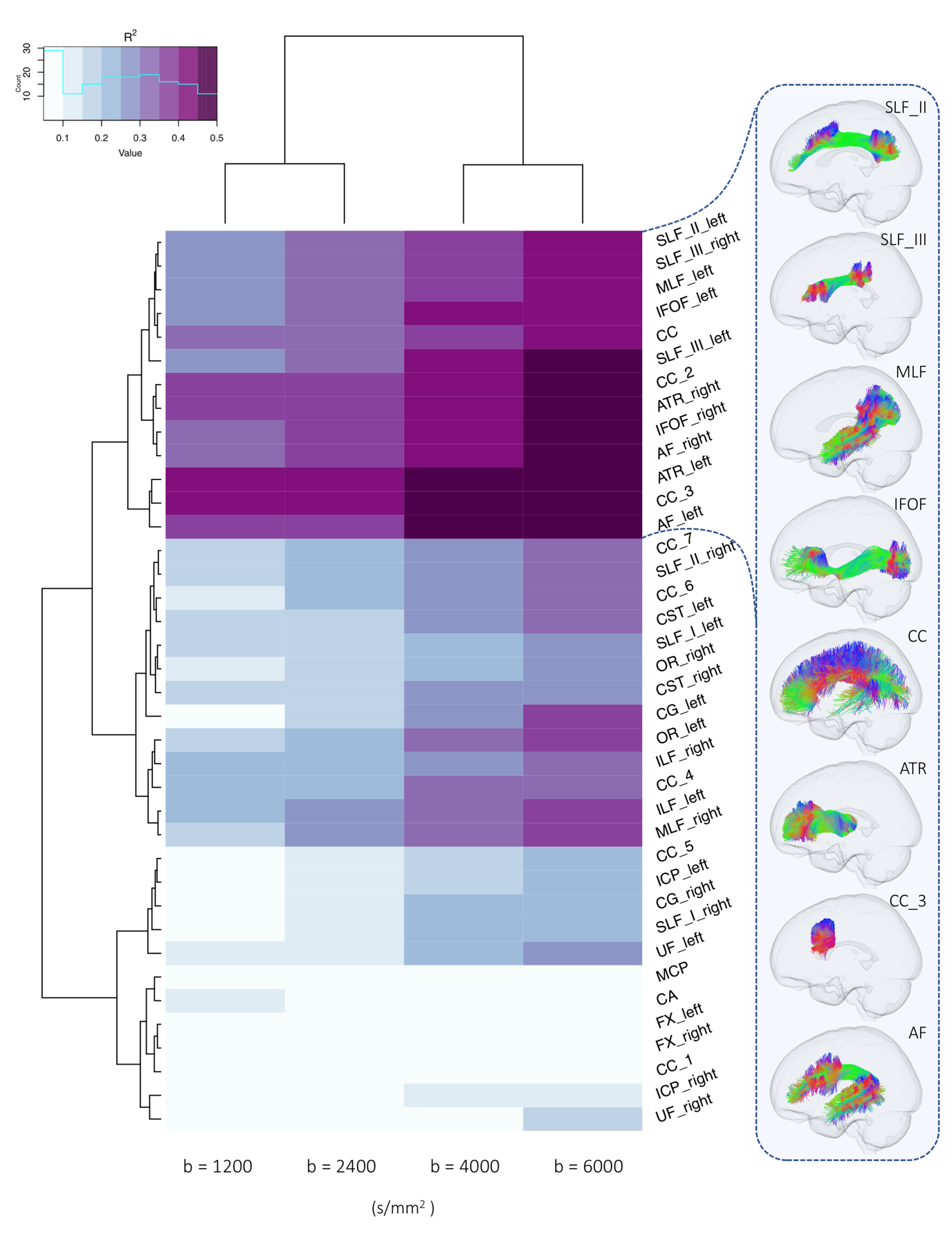


**Figure S4:** Dendrogram heatmap highlighting clusters of tracts which differentially describe age-related differences in apparent fibre density (AFD) across various single-shell b-value sampling schemes. Heatmap colour intensity reflects range of R^2^ values derived from a linear model including age and sex. Significant age-effects (*p_FWE_* < .05) are annotated with an asterisk (*). A depiction of several fibre pathways in one cluster is presented on the right.

**
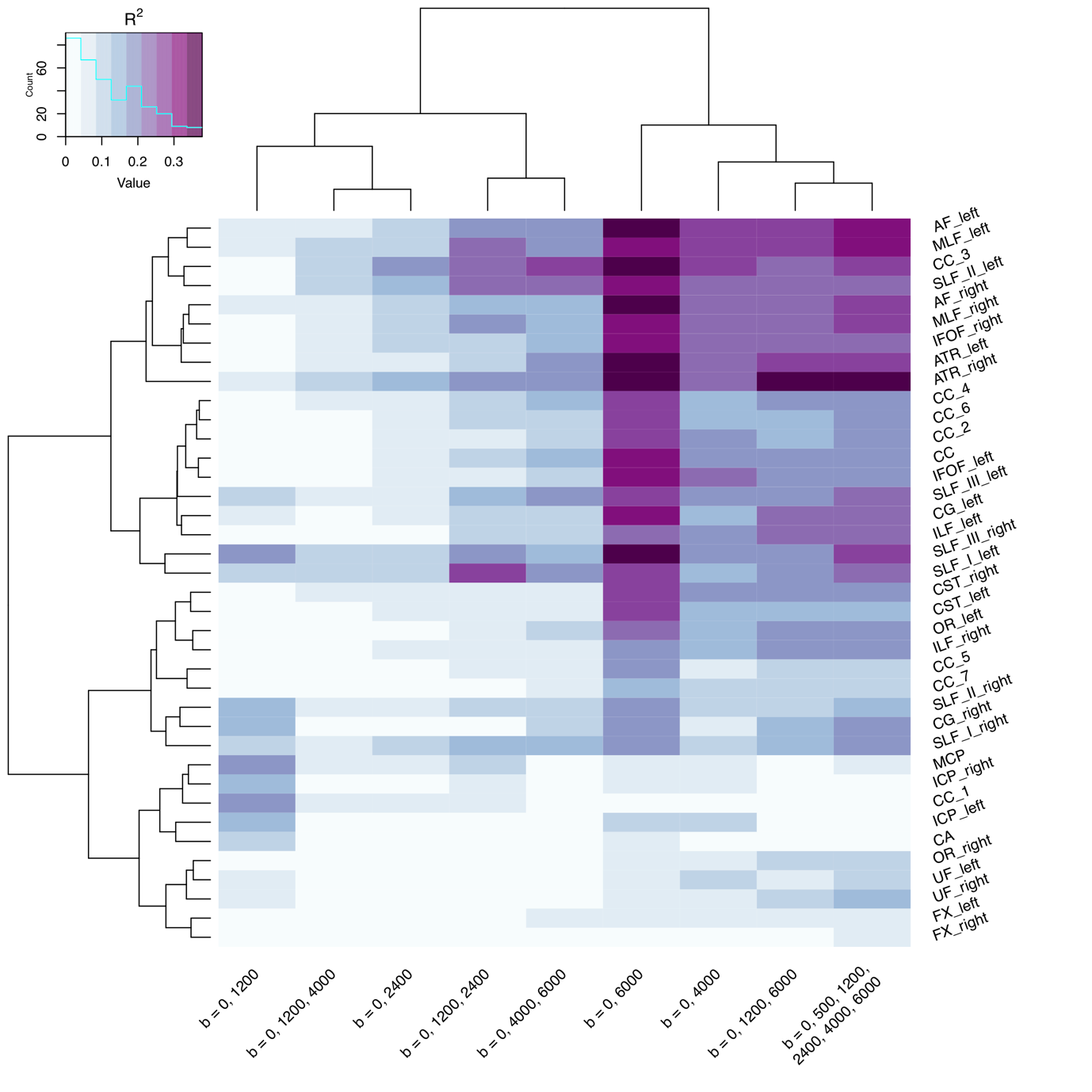
**

**Figure S5:** Dendrogram heatmap highlighting clusters of tracts which differentially describe age-related differences in apparent fibre density (AFD) across various multi-shell b-value sampling schemes. Heatmap colour intensity reflects range of R^2^ values derived from a linear model including age and sex. Note: datasets with two diffusion weightings (including the b=0) were processed using the multi-shell multi-tissue FBA framework, thus resulting in separate but comparable results to the single-shell single-tissue FBA (presented in Figure 3).

- 1. **Supplementary tables**

**Table S1:** Variance in AFD explained by age for a set of multi-shell sampling schemes across tracts.

| tracts | |  |  | R^2^ | | | |  | R^2^ Difference [95% CI] | | | | | | | |
| --- | --- | --- | --- | --- | --- | --- | --- | --- | --- | --- | --- | --- | --- | --- | --- | --- |
|  |  |  | ms_all_ | ms_6000_ | ms_4000_ | ms_2400_ | ms_1200_ |  | ms_6000_ > ms_4000_ | |  | ms_6000_ > ms_2400_ | |  | ms_6000_ > ms_1200_ | |
| AF | L |  | .32 | .35 | .29 | .09 | .08 |  | .16 | [.01, .16] |  | **.27** | [.14, .36] |  | **.27** | [.03, .51] |
|  | R |  | .29 | .36 | .23 | .11 | .08 |  | **.21** | [.07, .21] |  | **.25** | [.14, .38] |  | .28 | [-.06, .41] |
| ATR | L |  | .29 | .37 | .24 | .07 | .02 |  | **.25** | [.07, .25] |  | **.30** | [.24, .44] |  | **.36** | [.10, .51] |
|  | R |  | .39 | .38 | .25 | .14 | .07 |  | **.24** | [.08, .24] |  | **.24** | [.13, .34] |  | **.32** | [.14, .52] |
| CA |  |  | .02 | .05 | .01 | .02 | .10 |  | .14 | [-.06, .14] |  | .04 | [-.08, .12] |  | -.04 | [-.23, .11] |
| CC | full |  | .01 | .31 | .19 | .07 | .02 |  | **.21** | [.08, .21] |  | **.25** | [.16, .35] |  | **.30** | [.14, .50] |
|  | 1 |  | .17 | .01 | .02 | .06 | .19 |  | .03 | [-.20, .03] |  | -.06 | [-.15, .05] |  | -.19 | [-.36, .01] |
|  | 2 |  | .27 | .27 | .17 | .07 | .03 |  | **.19** | [.03, .19] |  | **.21** | [.10, .33] |  | **.24** | [.09, .45] |
|  | 3 |  | .17 | .37 | .25 | .17 | .04 |  | **.19** | [.06, .19] |  | **.20** | [.12, .28] |  | **.33** | [.19, .57] |
|  | 4 |  | .12 | .28 | .17 | .06 | .01 |  | **.19** | [.06, .19] |  | **.22** | [.13, .35] |  | **.27** | [.14, .45] |
|  | 5 |  | .19 | .18 | .08 | .03 | .02 |  | **.17** | [.05, .17] |  | **.15** | [.07, .30] |  | .17 | [-.08, .36] |
|  | 6 |  | .11 | .25 | .17 | .06 | .02 |  | **.15** | [.04, .15] |  | **.19** | [.09, .29] |  | **.23** | [.07, .43] |
|  | 7 |  | .20 | .13 | .12 | .03 | .01 |  | .15 | [-.04, .15] |  | **.09** | [.02, .20] |  | .12 | [-.08, .26] |
| CG | L |  | .25 | .30 | .14 | .05 | .05 |  | **.27** | [.08, .27] |  | **.25** | [.14, .45] |  | .25 | [-.10, .46] |
|  | R |  | .18 | .19 | .07 | .02 | .13 |  | **.21** | [.02, .21] |  | .17 | [-.01, .33] |  | .06 | [-.25, .28] |
| CST | L |  | .14 | .27 | .14 | .05 | .02 |  | **.20** | [.08, .20] |  | **.22** | [.10, .36] |  | .25 | [-.03, .39] |
|  | R |  | .17 | .28 | .18 | .08 | .02 |  | **.15** | [.05, .15] |  | **.19** | [.11, .30] |  | .26 | [.01, .43] |
| FX | L |  | .06 | .07 | .05 | .03 | .01 |  | .08 | [-.03, .08] |  | .05 | [-.02, .11] |  | .06 | [-.06, .15] |
|  | R |  | .04 | .04 | .02 | .01 | .02 |  | .07 | [-.03, .07] |  | .03 | [-.03, .11] |  | .02 | [-.08, .15] |
| ICP | L |  | .02 | .11 | .11 | .01 | .13 |  | .11 | [-.12, .11] |  | .11 | [.01, .33] |  | -.02 | [-.23, .25] |
|  | R |  | .03 | .06 | .05 | .03 | .15 |  | .09 | [-.07, .09] |  | .03 | [-.08, .18] |  | -.09 | [-.26, .12] |
| IFOF | L |  | .20 | .30 | .22 | .05 | .02 |  | **.16** | [.02, .16] |  | **.25** | [.14, .35] |  | **.28** | [.15, .44] |
|  | R |  | .25 | .32 | .23 | .10 | .02 |  | **.18** | [.04, .18] |  | **.23** | [.13, .36] |  | **.30** | [.08, .57] |
| ILF | L |  | .23 | .24 | .20 | .04 | .01 |  | .14 | [-.04, .14] |  | **.20** | [.10, .35] |  | **.23** | [.03, .46] |
|  | R |  | .21 | .18 | .13 | .05 | .01 |  | .13 | [-.04, .13] |  | **.13** | [.05, .25] |  | .18 | [-.01, .37] |
| MCP |  |  | .05 | .05 | .07 | .05 | .21 |  | .04 | [-.10, .04] |  | .00 | [-.14, .13] |  | -.16 | [-.46, -.03] |
| MLF | L |  | .31 | .31 | .26 | .11 | .04 |  | .14 | [.01, .14] |  | **.20** | [.11, .29] |  | **.27** | [.13, .46] |
|  | R |  | .27 | .33 | .22 | .10 | .04 |  | **.20** | [.06, .20] |  | **.24** | [.13, .33] |  | **.30** | [.07, .47] |
| OR | L |  | .20 | .21 | .14 | .04 | .04 |  | .17 | [.01, .17] |  | **.18** | [.02, .26] |  | .17 | [-.09, .36] |
|  | R |  | .10 | .07 | .07 | .01 | .02 |  | .10 | [-.03, .10] |  | .06 | [-.03, .16] |  | .05 | [-.06, .27] |
| SLF_III | L |  | .22 | .28 | .20 | .06 | .09 |  | **.17** | [.02, .17] |  | **.22** | [.13, .32] |  | .19 | [-.07, .44] |
|  | R |  | .26 | .35 | .20 | .12 | .19 |  | **.24** | [.10, .24] |  | **.23** | [.12, .37] |  | .16 | [-.18, .34] |
| SLF_II | L |  | .23 | .33 | .23 | .13 | .04 |  | **.14** | [.06, .14] |  | **.20** | [.14, .27] |  | **.29** | [.11, .46] |
|  | R |  | .13 | .17 | .11 | .06 | .14 |  | **.14** | [.03, .14] |  | .11 | [.01, .25] |  | .03 | [-.19, .25] |
| SLF_I | L |  | .23 | .29 | .16 | .11 | .09 |  | **.21** | [.08, .21] |  | **.18** | [.08, .29] |  | .21 | [-.04, .41] |
|  | R |  | .18 | .19 | .12 | .09 | .11 |  | .15 | [.01, .15] |  | .11 | [-.01, .22] |  | .08 | [-.15, .40] |
| UF | L |  | .09 | .06 | .09 | .01 | .05 |  | .07 | [-.11, .07] |  | .06 | [-.08, .19] |  | .01 | [-.26, .17] |
|  | R |  | .16 | .06 | .08 | .02 | .06 |  | .04 | [-.14, .04] |  | .04 | [-.04, .24] |  | .01 | [-.19, .24] |

*Note:* R^2^ represents the multiple coefficient of determination computed using a linear model for each tract, with age and sex as predictors. Difference indicates difference between R^2^ coefficients. Square brackets show 95% bias corrected accelerated (BCa) confidence intervals computed with 10,000 bootstrapped samples. Bold=differences in R^2^ where 0 was not captured by the confidence intervals. Abbreviations: AF: arcuate fasciculus; ATR: anterior thalamic radiation; CA: anterior commissure; CC: corpus callosum [1=rostrum, 2=genu, 3=rostral body, 4=anterior midbody, 5=posterior midbody; 6=isthmus, 7=splenium]; CG = cingulum; CST: corticospinal tract; FX: fornix; ICP: inferior cerebellar peduncle; IFOF: inferior fronto-occipital fasciculus; ILF: inferior longitudinal fasciculus; MCP: middle cerebellar peduncle; MLF: middle longitudinal fasciculus; OR: optic radiation; superior longitudinal fasciculus: SLF [I, II, III]; UF: uncinate fasciculus.
