# Supplementary figures and images for: Impact of b‐value on estimates of apparent fibre density
Source: Hum Brain Mapp. 2020 Mar 26;41(10):2583–95. doi: 10.1002/hbm.24964 (PMC7294071; doi:10.1002/hbm.24964)

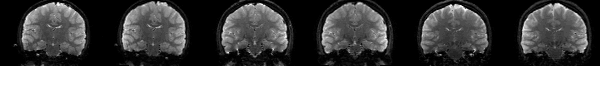

Supplement: Supplementary file 2 — Appendix S1: Supplementary Materials [file HBM-41-2583-s002.gif]
